# Instant orthopnea and pseudo‐cardiac dilatation due to anterior mediastinal tumor

**DOI:** 10.1002/jgf2.324

**Published:** 2020-04-29

**Authors:** Kiyoshi Shikino, Kazutaka Noda, Masatomi Ikusaka

**Affiliations:** ^1^ Department of General Medicine Chiba University Hospital Chiba‐City Japan

**Keywords:** Burkitt's lymphoma, instant orthopnea, mediastinal mass

## Abstract

A 45‐year‐old woman presented with instant orthopnea and enlarged cardiomediastinal silhouette in her chest radiograph. Although anterior mediastinal tumor can be misdiagnosed as heart failure due to orthopnea with enlarged cardiomediastinal silhouette, “instant orthopnea” may be a useful sign to distinguish these conditions.

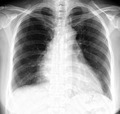

A 45‐year‐old Japanese woman presented with a 3‐week history of high‐grade fever, dyspnea, and anterior pleuritic chest pain. Her dyspnea was instantly exaggerated when in the supine position. Physical examination indicated bone tenderness with indirect pain at the anterior chest wall. Breath sounds were clear and there was no heart murmur. Laboratory test revealed high serum lactate dehydrogenase (1170 U/L) and serum‐soluble interleukin‐2 receptor (3658 U/L). Chest radiograph showed enlarged cardiomediastinal silhouette (Figure 1). Cardiac echocardiogram showed normal cardiac function but a large mass in the right ventricle. Chest computed tomography reveled mediastinal tumor invading anterior chest wall (Figure 2). Bone marrow biopsy exhibited Burkitt's lymphoma, and the patient was treated with CODOX‐M chemotherapy.

**Figure 1:**
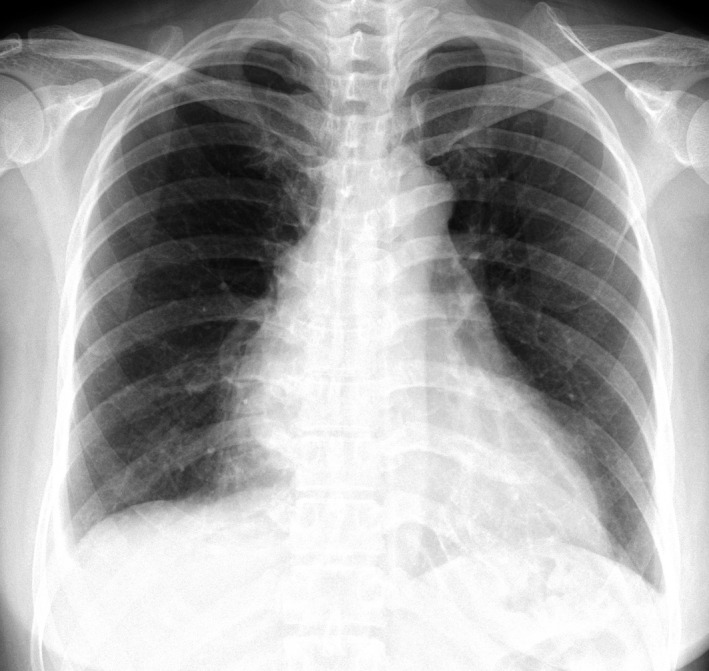
Chest radiograph showed enlarged cardiomediastinal silhouette

**Figure 2:**
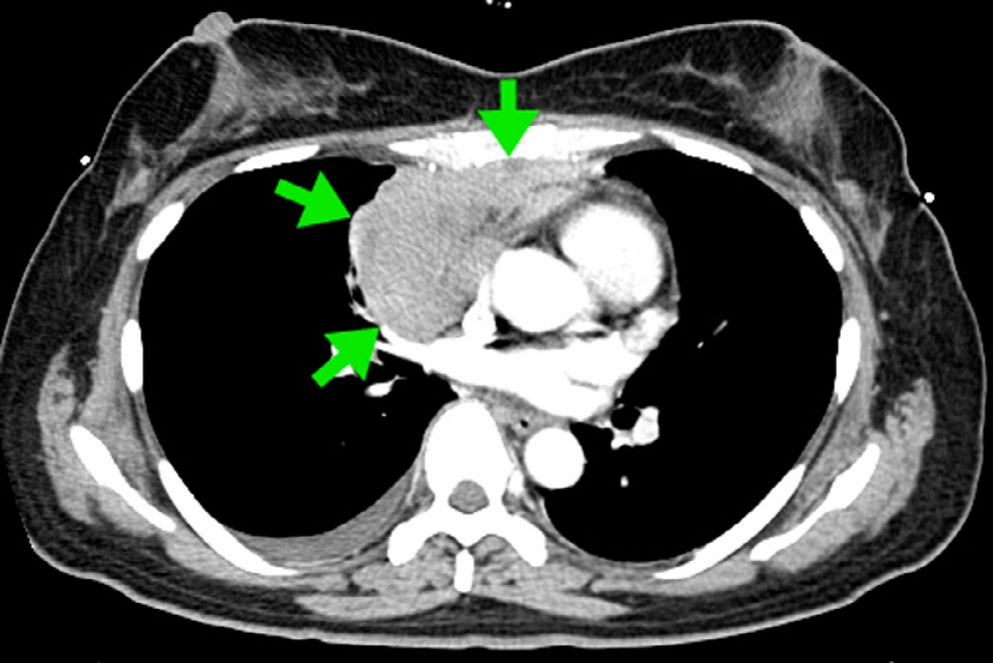
Chest computed tomography showed mediastinal tumor invading anterior chest wall (arrows)

Mediastinal tumors are usually asymptomatic. However, thoracic symptoms (chest pain, shortness of breath, dyspnea or orthopnea) may occur when the surrounding organs are pressed or infiltrated.^1^, ^2^ Orthopnea is commonly seen as a late manifestation of heart failure, which usually develops gradually over the course of the night. But our patient became orthopneic within minutes of recumbency, as seen in bilateral diaphragmatic paralysis.^3^ The mechanism of this “instant orthopnea” may be the direct compression of the anterior mass to the heart upon reclining. Although anterior mediastinal tumor can be misdiagnosed as heart failure due to orthopnea with enlarged cardiomediastinal silhouette, “instant orthopnea” may be a useful sign to distinguish these conditions.

## CONFLICT OF INTERESTS

The authors have stated explicitly that there are no conflicts of interest in connection with this article.
